# Clinical features and prognostic factors of patients with metastatic renal cell carcinoma stratified by age

**DOI:** 10.18632/aging.202637

**Published:** 2021-03-03

**Authors:** Gu Yue, Li Deyu, Tao Lianyuan, Shao Fengmin, Gao Mei, Huang Yajun, Zhang Wenwen, Yan Lei

**Affiliations:** 1 Department of Nephrology, Henan Provincial People’s Hospital, Henan Provincial Key Laboratory of Kidney Disease and Immunology, Zhengzhou University People’s Hospital, Henan University People’s Hospital, Zhengzhou 450003, Henan, China; 2 Department of Hepatobiliary Surgery, Henan Provincial People’s Hospital, People’s Hospital of Zhengzhou University, Henan University Peoples Hospital, Zhengzhou 450003, Henan, China

**Keywords:** renal cell carcinoma, metastasis, prognostic factors, elderly patients, survival

## Abstract

This study compared the clinicopathological characteristics and survival of patients with metastatic renal cell carcinoma (mRCC) stratified by age to identify clinical features and prognostic factors. Patients with renal cell carcinoma (RCC) between 2010 and 2015 were identified from the Surveillance, Epidemiology and End Results database. Age was an independent prognostic factor for patients with RCC, mRCC, mRCC of clear cell renal cell carcinoma and lung-related metastases. There were many significant differences between the younger and older groups, including differences in marital status, race, sex, year of diagnosis, histology grade, laterality, T stage, N stage, tumor size, type of treatment, including surgery, radiation or chemotherapy, and pattern of organic metastasis to the liver, lung, or brain (P<0.05). Moreover, different natural metastasis patterns and poorer overall survival were observed in the older group compared with the younger group (P<0.05). Parameters, including marital status, sex, year of diagnosis, histological grade, N stage, surgery, chemotherapy, lung metastasis and liver metastasis, were independent prognostic factors for elderly patients (P<0.05). Age plays a significant role in mRCC, and elderly patients with mRCC are a special group of individuals whose clinical characteristics and prognostic factors are different from those of younger patients; therefore, these patients require special attention.

## INTRODUCTION

Renal cell carcinoma (RCC) is a common urological malignancy with an increasing incidence in many areas [1–4], accounting for the twelfth most common cancer, with 337,860 cases recorded in 2012. Additionally, the incidence has been estimated to have increased by 22% to date [5]. Although advances in diagnostic techniques and surgical techniques have enabled earlier resection of early stage RCC, an increasing number of patients have distant metastases at the initial diagnosis, especially elderly patients [1, 3]. As the patient age increases, so does the risk of metastasis from RCC. Therefore, elderly patients with metastatic renal cell carcinoma (mRCC) are increasingly common and deserve attention.

Several studies have shown that tumors in elderly patients are unique, and the high proportion of tumors among elderly patients warrants further investigation of diagnostic and treatment practices [1, 3]. It is challenging to establish interdisciplinary collaborations to manage RCC in elderly patients and deliver the best possible care in the future [1]. Moreover, clinical characteristics and metastatic patterns have been indicated to be closely related to the prognosis of RCC [6–9]. Thus, accurately understanding the characteristics of patients with mRCC could help medical oncologists predict prognosis and provide treatment decisions. The present study explored the clinicopathological features and prognostic factors of patients with mRCC stratified by age.

## RESULTS

### Characteristics of patients with mRCC

**Figure 1:**
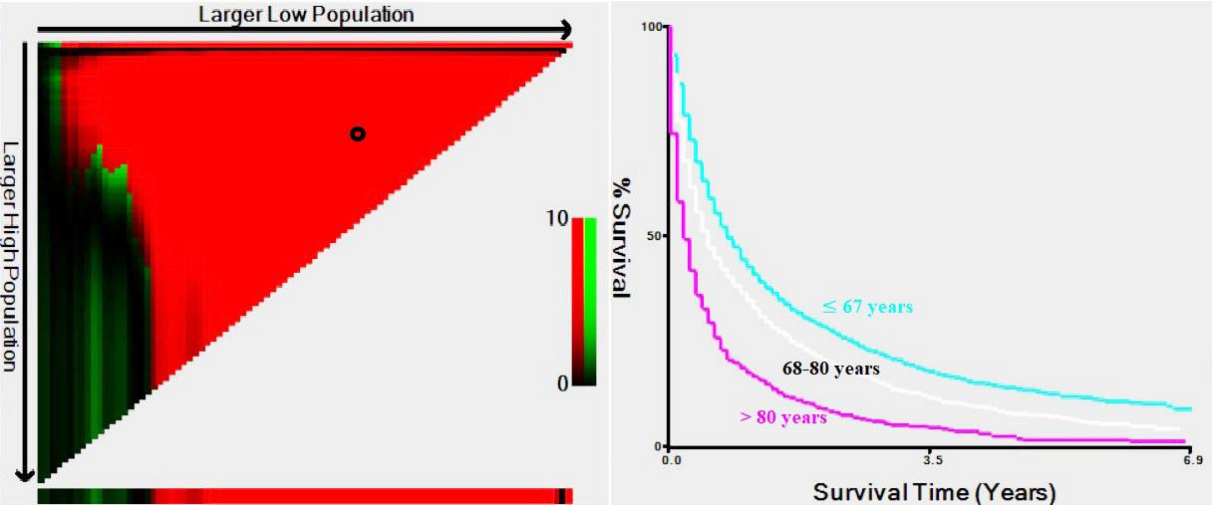
**Estimation of the cutoff values for age stratification, as determined by X-tile software.**

In total, 10,853 (13.7%) mRCC patients were selected among 79,063 patients with RCC. To better understand the metastasis patterns of RCC, we first compared the differences between patients with metastatic and nonmetastatic RCC (Supplementary Table 1). The results indicated that mRCC patients tended to be older, unmarried, white, and male and had a larger tumor size, higher grade, higher T stage and N stage, lower chance of undergoing radical nephrectomy or other operations and greater chance of receiving treatment, such as radiation and chemotherapy (P<0.001, Supplementary Table 1). Therefore, there were significant differences between the elderly patients and younger patients. We then used X-tile software to divide the patients by age into three groups (Figure 1). There were 49,801 diagnosed RCC patients ≤67 years old, and they were classified as the younger group; 29,262 (68-80 years old) patients were classified as the middle-aged group, and 6,492 patients were included in the older group (>80 years old). Moreover, among the total population of 10,853 mRCC patients, 6,131 (56.4%) were in the younger group, 3,255 (30.0%) were in the middle-aged group, and 1,467 (13.5%) were in the older group (>80 years old). The younger group had the lowest metastasis rate of 12.3% (6,131/49,801), the middle-aged group had a rate of 11.1% (3,255/29,262), and the older group had the highest metastasis rate of 22.6% (1,467/6,492).

Clear cell RCC was found to be the most common histological type (42,702). However, 16,990 (21.5%) patients had no definite histological diagnosis. If these patients were not counted, then the proportion of clear cell RCC was 68.8% (42,702/62,032). The tumor size was also divided into three groups using X-tile software (Supplementary Figure 1). The lung was the most common metastatic site (6,589, 60.7%), followed by bone (4,233, 39%), liver (2,339, 21.6%), and brain (1,182, 10.9%). Of the 10,853 mRCC patients, mortality occurred in 9,014 (83.1% of 10,853) patients by the end of the follow-up period.

We then performed a univariate analysis of the patients with mRCC to identify significant variables associated with survival (Supplementary Table 2). Our data showed that marital status, age, race, sex, year of diagnosis, histological grade, T stage, N stage, surgery, chemotherapy, metastasis site (lung, bone, liver or brain), and tumor size were prognostic factors for OS (P<0.05). Multivariate Cox regression analysis revealed that marital status, age, year of diagnosis, histological grade, T stage, N stage, metastasis site (lung, bone, liver, or brain), surgical resection, chemotherapy, and tumor size were independent prognostic factors (Supplementary Table 2, P<0.05). Therefore, age was significantly associated with the OS of patients with mRCC and is an independent prognostic factor for mRCC.

### Multivariate analysis of factors affecting OS in mRCC patients with clear cell RCC

Regarding the histological types of mRCC, clear cell RCC (4,229 cases) was the most common type. It has been indicated that histological subtypes follow distinct clinical courses and have varying treatment responses [10]. We then performed a multivariate analysis of the mRCC patients with clear cell RCC to identify significant factors associated with survival (Supplementary Table 3). Our data showed that marital status, sex, year of diagnosis, histological grade, N stage, surgery, chemotherapy, metastasis site (lung, bone, liver, and brain), and tumor size were independent prognostic factors, similar to the factors for mRCC (P <0.05, Supplementary Table 3). Most importantly, age was also an independent prognostic factor for patients with metastatic clear cell RCC.

### Multivariate analysis of factors affecting OS rates in patients with lung-related metastases

Since the lung was the most common metastatic site for mRCC, we further performed Cox regression analysis to identify significant factors associated with survival for mRCC patients with lung-related metastases. Our data revealed that marital status, year of diagnosis, histological grade, T stage, N stage, metastasis site (lung, bone brain or liver), surgical resection, chemotherapy, and tumor size were independent prognostic factors, similar to the factors for mRCC (P<0.05, Supplementary Table 4). Above all, age was also an independent prognostic factor for mRCC patients with lung-related metastases.

### Analysis of patients with mRCC stratified by age

Since age was an independent prognostic factor for patients with RCC, mRCC, mRCC of clear cell RCC and lung-related metastases, age plays a significant role in mRCC. To better understand the role of age in mRCC, we performed a further study of patients with mRCC stratified by age by comparing the clinical features of different age groups. Compared with the younger group, the older group showed many significant differences, including differences in marital status, race, sex, year of diagnosis, histology grade, literality, T stage, N stage, tumor size, type of treatment, including surgery, radiation or chemotherapy, and organic metastasis pattern to the liver, lung, or brain (P<0.05, Table 1).

**Table 1:** Clinical characteristics of renal cell carcinoma with distant metastasis among different age group.

		**Total**	**Percentage**	**Age ≤ 67 years**	**Percentage**	**68-80 years**	**Percentage**	**Age > 82 years**	**Percentage**	**P-value**
		10853	100.00%	6131	100.00%	3255	100.00%	1467	100.00%	
**Marital status**	Married	6067	55.90%	3490	56.90%	1960	60.20%	617	42.10%	<0.001
	Unmarried	4339	40.00%	2376	38.80%	1173	36.00%	790	53.90%	
	Unknown	447	4.10%	265	4.30%	122	3.70%	60	4.10%	
**Race**	White	8938	82.40%	4903	80.00%	2765	84.90%	1270	86.60%	<0.001
	Black	1137	10.50%	756	12.30%	279	8.60%	102	7.00%	
	Other	778	7.20%	472	7.70%	211	6.50%	95	6.50%	
**Sex**	Male	7358	67.80%	4400	71.80%	2129	65.40%	829	56.50%	<0.001
	Female	3495	32.20%	1731	28.20%	1126	34.60%	638	43.50%	
**Year of diagnosis**	2010-2012	5166	47.60%	2985	48.70%	1490	45.80%	691	47.10%	0.025
	2013-2015	5687	52.40%	3146	51.30%	1765	54.20%	776	52.90%	
**Histological grade**	I-II	1045	9.60%	644	10.50%	329	10.10%	72	4.90%	<0.001
	III-IV	3384	31.20%	2292	37.40%	895	27.50%	197	13.40%	
	Unknown	6424	59.20%	3195	52.10%	2031	62.40%	1198	81.70%	
**Laterality**	Left	5285	48.70%	3045	49.70%	1556	47.80%	684	46.60%	<0.001
	Right	5003	46.10%	2833	46.20%	1505	46.20%	665	45.30%	
	Other	565	5.20%	253	4.10%	194	6.00%	118	8.00%	
**T**	≤T1	2081	19.20%	988	16.10%	692	21.30%	401	27.30%	<0.001
	T2	1740	16.00%	1046	17.10%	489	15.00%	205	14.00%	
	T3	3505	32.30%	2251	36.70%	999	30.70%	255	17.40%	
	T4	1388	12.80%	843	13.70%	388	11.90%	157	10.70%	
	TX	2139	19.70%	1003	16.40%	687	21.10%	449	30.60%	
**N**	N0	6034	55.60%	3362	54.80%	1863	57.20%	809	55.10%	<0.001
	N1	3364	31.00%	2059	33.60%	914	28.10%	391	26.70%	
	NX	1455	13.40%	710	11.60%	478	14.70%	267	18.20%	
**Tumor size**	≤45mm	1569	14.50%	734	12.00%	534	16.40%	301	20.50%	<0.001
	46-80mm	3090	28.50%	1582	25.80%	1027	31.60%	481	32.80%	
	> 80mm	4800	44.20%	3182	51.90%	1247	38.30%	371	25.30%	
	Unknown	1394	12.80%	633	10.30%	447	13.70%	314	21.40%	
**Surgery**	No/Unknown	7086	65.30%	3474	56.70%	2288	70.30%	1324	90.30%	<0.001
	Radical nephrectomy	2997	27.60%	2136	34.80%	758	23.30%	103	7.00%	
	Other operation	770	7.10%	521	8.50%	209	6.40%	40	2.70%	
**Radiation**	No/Unknown	7950	73.30%	4236	69.10%	2463	75.70%	1251	85.30%	<0.001
	Yes	2903	26.70%	1895	30.90%	792	24.30%	216	14.70%	
**Chemotherapy**	No/Unknown	5699	52.50%	2714	44.30%	1811	55.60%	1174	80.00%	<0.001
	Yes	5154	47.50%	3417	55.70%	1444	44.40%	293	20.00%	
**Metastasis at bone**	No	6620	61.00%	3684	60.10%	2019	62.00%	917	62.50%	0.082
	Yes	4233	39.00%	2447	39.90%	1236	38.00%	550	37.50%	
**Metastasis at brain**	No	9671	89.10%	5318	86.70%	2969	91.20%	1384	94.30%	<0.001
	Yes	1182	10.90%	813	13.30%	286	8.80%	83	5.70%	
**Metastasis at liver**	No	8514	78.40%	4830	78.80%	2559	78.60%	1125	76.70%	0.207
	Yes	2339	21.60%	1301	21.20%	696	21.40%	342	23.30%	
**Metastasis at lung**	No	4264	39.30%	2303	37.60%	1320	40.60%	641	43.70%	<0.001
	Yes	6589	60.70%	3828	62.40%	1935	59.40%	826	56.30%	

To explore the natural metastasis patterns among mRCC patients stratified by age, we identified 9,581 patients with certain metastatic sites (lung, bone, brain, and bone). Regarding the observation of metastatic sites in these RCC patients, a single lung metastasis was the most common, followed by metastasis to the bone, liver, and brain, in all age groups. In patients with multiple metastatic sites, lung-related metastases occurred most in the lung + bone, followed by lung + liver, in all age groups (P<0.05, Table 2). The comparison of different age groups showed that with increasing age, single metastases and certain combinations of two sites of metastasis (bone and brain, bone, and liver) occurred less frequently; in contrast, other combinations of two sites of metastases and almost all combinations of three sites of metastases showed an increasing trend with age (P<0.05, Table 2).

**Table 2:** Comparison of organ metastasis patterns stratified by age patients with metastatic renal cell carcinoma.

**Parameter**	**≤ 67 years**	**Percentage**	**68-80 years**	**Percentage**	**> 80 years**	**Percentage**	**P-value**
	n=5620	100.0%	n=2808	100.0%	n=1153	100.0%	
**Lung metastasis only**	1923	34.2%	943	33.6%	379	32.9%	0.631
**bone metastasis only**	1278	22.7%	398	14.2%	79	6.9%	<0.001
**Brain metastasis only**	152	2.7%	72	2.6%	18	1.6%	0.078
**Liver metastasis only**	384	6.8%	168	6.0%	53	4.6%	0.012
**Lung and brain**	171	3.0%	121	4.3%	86	7.5%	<0.001
**Lung and bone**	644	11.5%	449	16.0%	190	16.5%	<0.001
**Bone and brain**	85	1.5%	12	0.4%	4	0.3%	<0.001
**Bone and liver**	176	3.1%	59	2.1%	20	1.7%	0.002
**Lung and liver**	333	5.9%	260	9.3%	169	14.7%	<0.001
**Brain and liver**	7	0.1%	13	0.5%	4	0.3%	0.011
**Lung, bone and brain**	110	2.0%	88	3.1%	40	3.5%	<0.001
**Lung, bone and liver**	250	4.4%	157	5.6%	87	7.5%	<0.001
**Lung, brain and liver**	42	0.7%	26	0.9%	24	2.1%	<0.001
**Bone, brain, and liver**	10	0.2%	0	0.0%	0	0.0%	0.029
**Lung, bone, brain, and liver**	55	1.0%	42	1.5%	0	0.0%	<0.001

**Figure 2:**
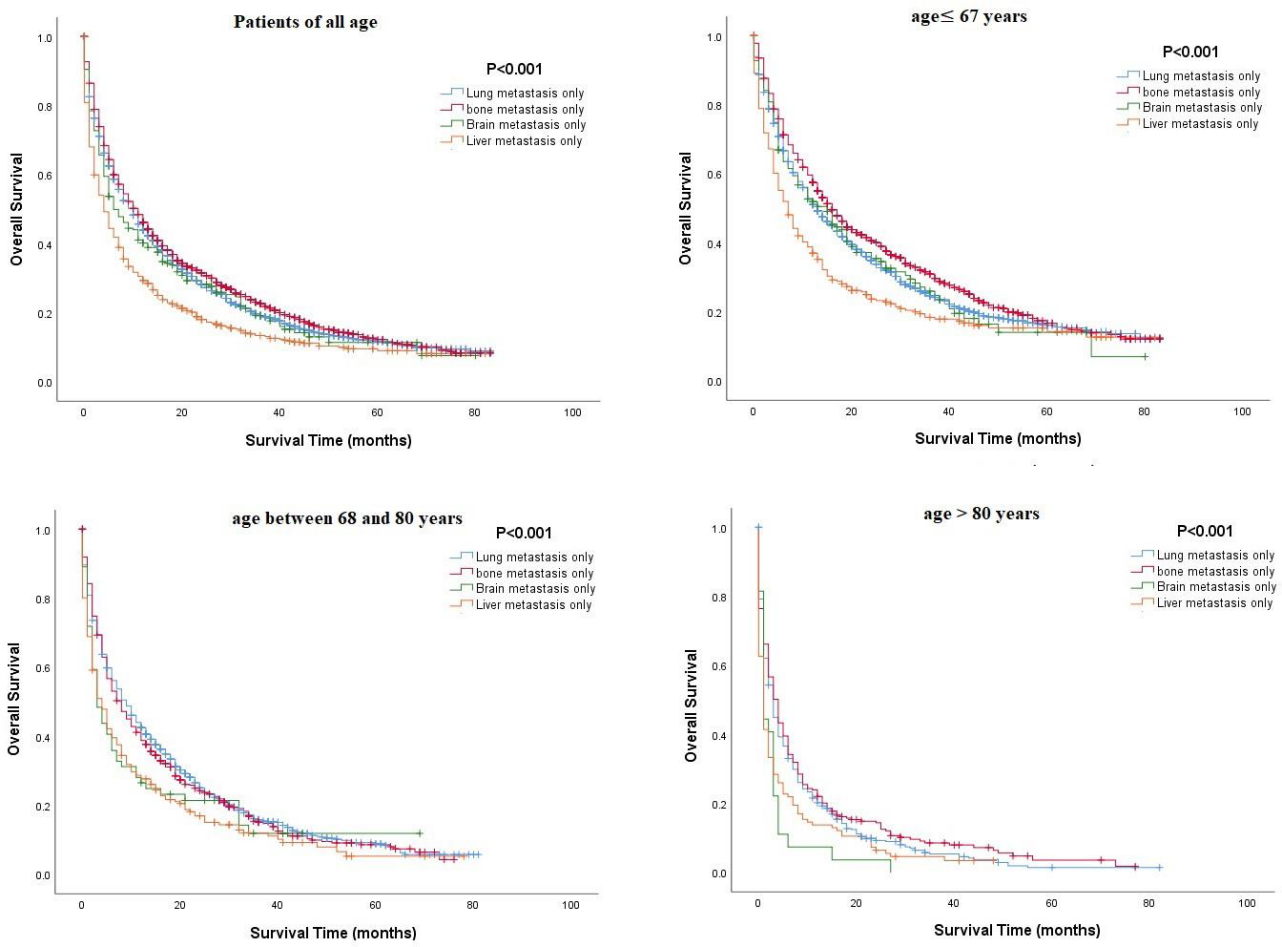
**Comparison of overall survival rates among patients with renal cell carcinoma and a single metastatic site in different age groups.**

**Figure 3:**
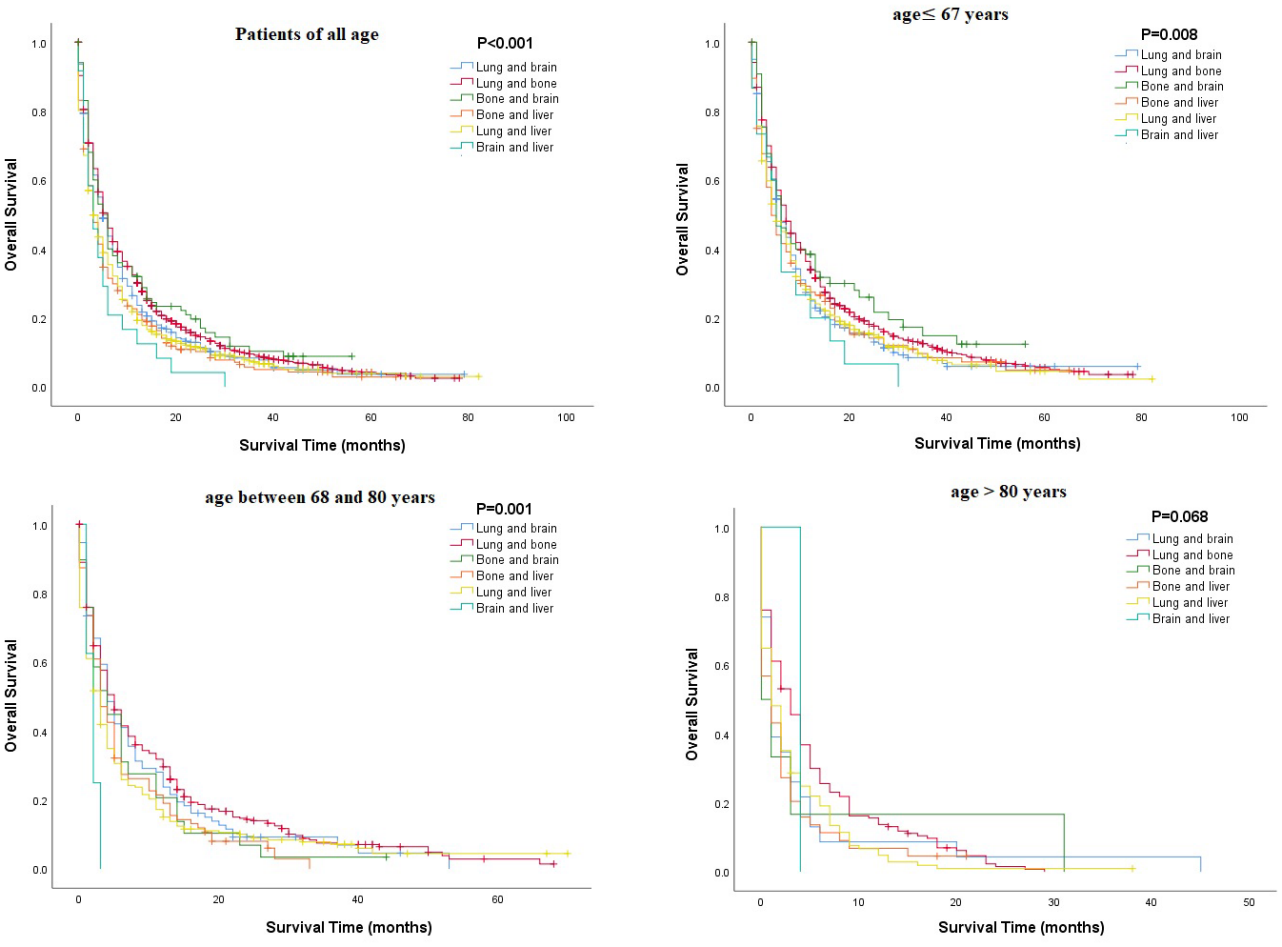
**Comparison of overall survival rates among patients with renal cell carcinoma and two metastatic sites in different age groups.**

Multivariate Cox regression analysis of mRCC among different age groups revealed that several parameters, such as being unmarried, having a higher grade or N stage, and having metastasis (lung or brain), were associated with poorer OS in all age groups, while surgical resection or chemotherapy was associated with better OS (P<0.05, Table 3). Unlike other age groups, in the older group, being unmarried and male were related to poor OS (Table 3). Kaplan-Meier analysis was performed to compare the OS rates of patients with RCC and a single metastatic site in different age groups. The results showed that in all age groups, patients with metastasis to the lung or bone tended to have better OS, and those with metastasis to the liver had poorer OS (Figure 2). Moreover, Kaplan-Meier analysis for those with two metastatic sites indicated that patients with metastasis to the lung + bone and bone + brain had better OS than those with metastasis to the brain + liver (P<0.05, Figure 3).

**Table 3:** Multivariate Cox regression analysis of overall survival (OS) rates of the metastasis by age groups.

		**≤ 67 years**		**68-80 years**		**> 80 years**	
		HRs (95% CI)	P-value	HRs (95% CI)	P-value	HRs (95% CI)	P-value
**Marital status**	Married	1 (Ref)		1 (Ref)		1 (Ref)	
	Unmarried	1.10 (1.03-1.16)	0.003	1.07 (0.99-1.17)	0.092	1.17 (1.04-1.32)	0.010
	Unknown	0.79 (0.68-0.92)	0.002	1.20 (0.98-1.47)	0.074	0.88 (0.67 -1.17)	0.386
**Race**	White	1 (Ref)		1 (Ref)		1 (Ref)	
	Black	1.08 (0.99-1.18)	0.090	0.96 (0.84-1.10)	0.602	0.96 (0.78-1.19)	0.723
	Other	0.91 (0.81-1.01)	0.073	0.96 (0.82-1.12)	0.616	1.05 (0.84-1.31)	0.654
**Sex**	Male	1 (Ref)		1 (Ref)		1 (Ref)	
	Female	0.99 (0.93-1.06)	0.842	1.12 (1.03-1.21)	0.009	0.88 (0.78-0.99)	0.032
**Year of diagnosis**	2010-2012	1 (Ref)		1 (Ref)		1 (Ref)	
	2013-2015	0.91 (0.86-0.97)	0.002	0.95 (0.88-1.03)	0.193	0.98 (0.88-1.09)	0.726
**Histological grade**	I-II	1 (Ref)		1 (Ref)		1 (Ref)	
	III-IV	1.54 (1.37-1.73)	<0.001	1.31 (1.13-1.52)	<0.001	1.42 (1.04-1.93)	0.026
	Unknown	1.30 (1.15-1.45)	<0.001	1.21 (1.05-1.41)	0.010	1.26 (0.95-1.66)	0.107
**Laterality**	Left	1 (Ref)		1 (Ref)		1 (Ref)	
	Right	0.97 (0.92 -1.03)	0.302	0.97 (0.90-1.05)	0.454	1.03 (0.92-1.15)	0.575
	Unknown	0.96 (0.82-1.12)	0.582	0.89 (0.75-1.06)	0.192	1.06 (0.84-1.32)	0.636
**T**	≤T1	1 (Ref)		1 (Ref)		1 (Ref)	
	T2	1.07 (0.95-1.21)	0.278	0.97 (0.83-1.13)	0.670	1.08 (0.86-1.34)	0.513
	T3	1.14 (1.02-1.27)	0.021	1.11 (0.97-1.27)	0.125	1.16 (0.96-1.41)	0.130
	T4	1.31 (1.16-1.48)	<0.001	1.24 (1.06-1.44)	0.008	1.10 (0.88-1.37)	0.406
	TX	1.13 (0.99-1.28)	0.062	1.00 (0.87-1.15)	0.986	1.09 (0.91-1.30)	0.363
**N**	N0	1 (Ref)		1 (Ref)		1 (Ref)	
	N1	1.51 (1.41-1.61)	<0.001	1.46 (1.33-1.59)	<0.001	1.22 (1.07-1.39)	0.002
	NX	1.11 (1.01-1.23)	0.038	1.17 (1.04-1.32)	0.007	1.07 (0.91-1.26)	0.385
**Surgery**	No/Unknown	1 (Ref)		1 (Ref)		1 (Ref)	
	Radical nephrectomy	0.41 (0.38-0.45)	<0.001	0.43 (0.37-0.48)	<0.001	0.43 (0.32-0.57)	<0.001
	Other operation	0.40 (0.35-0.45)	<0.001	0.41 (0.34-0.49)	<0.001	0.44 (0.31-0.63)	<0.001
**Radiation**	No/Unknown	1 (Ref)		1 (Ref)		1 (Ref)	
	Yes	1.02 (0.95-1.10)	0.576	0.94 (0.84-1.04)	0.226	0.95 (0.80-1.12)	0.534
**Chemotherapy**	No/Unknown	1 (Ref)		1 (Ref)		1 (Ref)	
	Yes	0.67 (0.64-0.72)	<0.001	0.59 (0.54-0.64)	<0.001	0.59 (0.51-0.68)	<0.001
**Metastasis at bone**	No	1 (Ref)		1 (Ref)		1 (Ref)	
	Yes	1.22 (1.15-1.31)	<0.001	1.35 (1.23-1.48)	<0.001	1.12 (0.99-1.27)	0.072
**Metastasis at brain**	No	1 (Ref)		1 (Ref)		1 (Ref)	
	Yes	1.41 (1.29-1.55)	<0.001	1.44 (1.26-1.65)	<0.001	1.22 (0.97-1.54)	0.092
**Metastasis at liver**	No	1 (Ref)		1 (Ref)		1 (Ref)	
	Yes	1.41 (1.32-1.52)	<0.001	1.51 (1.37-1.65)	<0.001	1.30 (1.14-1.48)	<0.001
**Metastasis at lung**	No	1 (Ref)		1 (Ref)		1 (Ref)	
	Yes	1.39 (1.31-1.48)	<0.001	1.27 (1.17-1.38)	<0.001	1.20 (1.07-1.35)	0.002
**Tumor size**	≤45mm	1 (Ref)		1 (Ref)		1 (Ref)	
	46-80mm	0.99 (0.89-1.10)	0.822	1.21 (1.07-1.37)	0.003	1.05 (0.89-1.23)	0.582
	> 80mm	1.11 (0.99-1.25)	0.065	1.24 (1.08-1.43)	0.003	1.18 (0.96-1.45)	0.116
	Unknown	1.00 (0.87-1.15)	0.992	1.30 (1.10-1.54)	0.002	1.00 (0.81-1.23)	0.999

Abbreviations: HR=Hazard ratio; CI=confidence interval.

## DISCUSSION

The incidence of RCC has increased rapidly, and its prognosis is inversely associated with metastasis [8]. Our data show that approximately 13.7% (10,853/79,060) of patients with RCC have visceral metastases. It has been reported that approximately 15-18% of patients present with mRCC at diagnosis, which is similar to our data [11, 12]. In addition, up to 40% of patients eventually develop metastatic disease during follow-up [11, 12]. With the increasing aging population, the population of patients with mRCC is also increasing, which is supported by the present study. Our data show that the older group had the highest metastasis rate of 22.6%, which is drastically higher than that in the younger group (12.3%) and the middle-aged group (11.1%).

Distant metastases occur most often in the lungs (60-75%), liver (19-40%), bone (39-40%), and brain (5-7%) [13]. The current study found a similar result: lung metastasis was the most common (60.7%), followed by metastasis to the bone (39%), liver (21.6%), and brain (10.9%). Furthermore, in cases of mRCC with multiple metastatic sites, lung-related metastases occurred most in the lung + bone. This trend was similar in all age groups. The reason for the observed high incidence of RCC metastasis to the lung could be attributed to the immune landscape reshaped by cancer cells through the secretion of cytokines or chemokines, which trigger neutrophil-dependent lung metastasis [14]. The “seed and soil hypothesis” may also partly explain the phenomenon of different metastasis sites and patterns of metastasis [15]. Our study shows that patients with lung metastasis tended to have better OS than patients with other sites of metastases or multiple metastatic sites. RCC usually metastasizes through the bloodstream, and the lung is the first organ to be affected. Cancer cells spreading from the bloodstream to other organs, such as the bone, brain, or liver, represent a more advanced stage of cancer or a tumor that is capable of subsequent invasion and metastasis. This may account for this poor prognosis of patients with metastasis to these organs, especially those with multiple sites of metastases. Our study indicates that age is an independent prognostic factor for patients with RCC, mRCC, mRCC of clear cell RCC and lung-related metastases. Consequently, to further investigate the role of age in mRCC, we selected the cutoff value through X-tile software. The ages of 80 and 67 years were chosen as the optimal values, and patients aged younger than 67 years showed the best prognosis.

We first focused on the demographic data. Our results showed that patients in the older group were more likely to be unmarried, which may be because elderly patients have a greater chance of losing their mate. In addition, the proportion of patients between 68 and 80 years old who were married was the highest (1,960/3,255, 60.2%) among all age groups, while the proportion of married patients above 80 years old was sharply reduced (617/1,467, 42.1%), which may be attributed to the higher death rate of spouses in this age range. Further prognostic analysis indicated that marriage is a favorable prognostic factor for mRCC patients above 80 years old, which agrees with many previous studies [16–18]. The results indicate that elderly people who lack care from a marriage partner have a poor prognosis. Therefore, elderly patients who do not have spouses should be given more attention. Moreover, the present study indicated that the proportion of whites increased with age, which may be attributed to the fact that white patients are more likely to have a longer life span, possibly because white patients tend to have more access to medical services [19, 20]. In addition, although our study showed that men comprised the majority, the proportion of women increased with age, which may be because females tend to pay more attention to their health and make greater use of healthcare services than males [21, 22]. The survival analysis of this study further demonstrated that being female was independent prognostic factor for elderly patients with mRCC.

Regarding clinicopathological data, our results suggested that patients in the older group had more T1 stage disease, less N1 stage disease, a smaller tumor size, and a lower chance for metastasis to the lung and brain than patients in the younger group, but they had a higher chance of developing liver metastases. This result can be attributed to the frequent routine check-ups of elderly patients, which can allow for the diagnosis of disease at an early stage. Moreover, prognostic factor analysis suggested that patients with N1 stage disease or with metastasis to the liver tended to have a poorer prognosis, which may further support the suggestion that older patients have a poorer prognosis. These results are in agreement with age-stratified analyses of other cancers [23–25].

Data on treatment indicated that the elderly have a lower chance of receiving treatments, such as surgery, radiation, and chemotherapy. To date, the treatment selected for RCC, including surgery, chemotherapy or radiotherapy, depends on the stage of the disease; however, age should also be taken into account [6–8, 11, 26, 27]. Further analysis indicated that surgery and chemotherapy were independent prognostic factors for elderly patients, which is in agreement with previous studies of other cancers [23–25]. Moreover, treatments, such as surgery or chemotherapy, have already been proven to be prognostic factors for renal cancer [6, 7, 11, 28]. Regarding other clinical characteristics, such as the year of diagnosis and laterality, although there were significant differences between the elderly group and the two younger groups, there was no trend of change with age. We can conclude that there is a difference between elderly and younger patients, but we cannot judge the relationship of this difference between the trend of change and age.

According to our results, elderly patients with mRCC are a special group of individuals whose clinical characteristics and prognostic factors are different from those of patients in other age groups. Therefore, more individualized attention should be paid to elderly mRCC patients to improve their survival rate and quality of life. However, our research does have some limitations. First, due to the retrospective nature of the present analysis, selection bias may have been present. Second, we were unable to collect detailed data on systematic treatment or other variables related to treatment regimens, such as quantity and exact location, from the SEER database. Thus, we could not evaluate the contribution of these factors or their survival benefits. Third, the SEER database started providing data about the location of distant metastasis in 2010, and the most recent data about tumor size were from 2015. Therefore, only patients from 2010 to 2015 were involved. Despite the stated limitations, our study is a population-based study that included a large number of mRCC patients, and the results are convincing.

## CONCLUSIONS

Age plays a significant role in mRCC, and elderly patients with mRCC are a special group of individuals whose clinical characteristics and prognostic factors are different from those of younger patients. These patients therefore require special attention.

## MATERIALS AND METHODS

### Patient cohort

The data examined in our study were retrieved from the Surveillance Epidemiology and End Results (SEER) database. In this study, we utilized SEER* Stat 8.3.5 software to query data from 18 SEER registries. In total, 85,381 patients were identified with a primary site of ‘kidney’ between January 1, 2010 and December 31, 2015, and 11,490 patients were considered to have American Joint Committee on Cancer (AJCC) (7^th^ edition) stage IV disease [29]. After excluding patients with unknown sites of cancer metastasis, an unknown age or race, or who lacked survival data, 79,063 patients remained (10,853 with stage IV disease). As a publicly available database, the SEER database contains deidentified data; therefore, this study did not need approval from the institutional review board.

### Data collection

The following information was collected from each patient: marital status, age, race, sex, year of diagnosis, primary site of the tumor, T stage, N stage, M stage, surgical resection of the primary tumor, chemotherapy recode, tumor size, survival time, and vital status. Overall survival (OS) was defined as the time between diagnosis and death from any cause. Detailed information on systematic treatment is not available in the SEER database. Histological subtypes of mRCC in the following statistical analyses are based on the third edition ICD-O-3 codes. Clear cell renal cell carcinoma (8310/3) was the most common type in the SEER database, and a separate statistical analysis will be performed for this subtype.

### Statistical analyses

X-tile software v3.6.1 (Yale University, New Haven, CT, USA) was utilized to determine the optimal cutoff values for age [30]. Clinical and demographic features were compared with the chi-square test. The Kaplan-Meier method with the log-rank test was used to assess OS. A Cox proportional hazards model was applied for multivariable survival analysis of OS. The hazards ratio (HR) and 95% confidence interval (95% CI) were also generated for statistically significant variables. P<0.05 was considered statistically significant. IBM SPSS Statistics 25.0 (IBM, Armonk, NY, USA) was applied for all statistical analyses.

### Ethical approval

This article does not contain any studies with human participants or animals performed by any of the authors.

## Supplementary Material

Supplementary Figure 1

Supplementary Tables
