# Supplementary material for: Clinical features and prognostic factors of patients with metastatic renal cell carcinoma stratified by age
Source: Aging (Albany NY). 2021 Mar 3;13(6):8290–305. doi: 10.18632/aging.202637 (PMC8034891; doi:10.18632/aging.202637)
Supplement: Supplementary Figure 1 [file aging-13-202637-s001.pdf]

SUPPLEMENTARY FIGURE

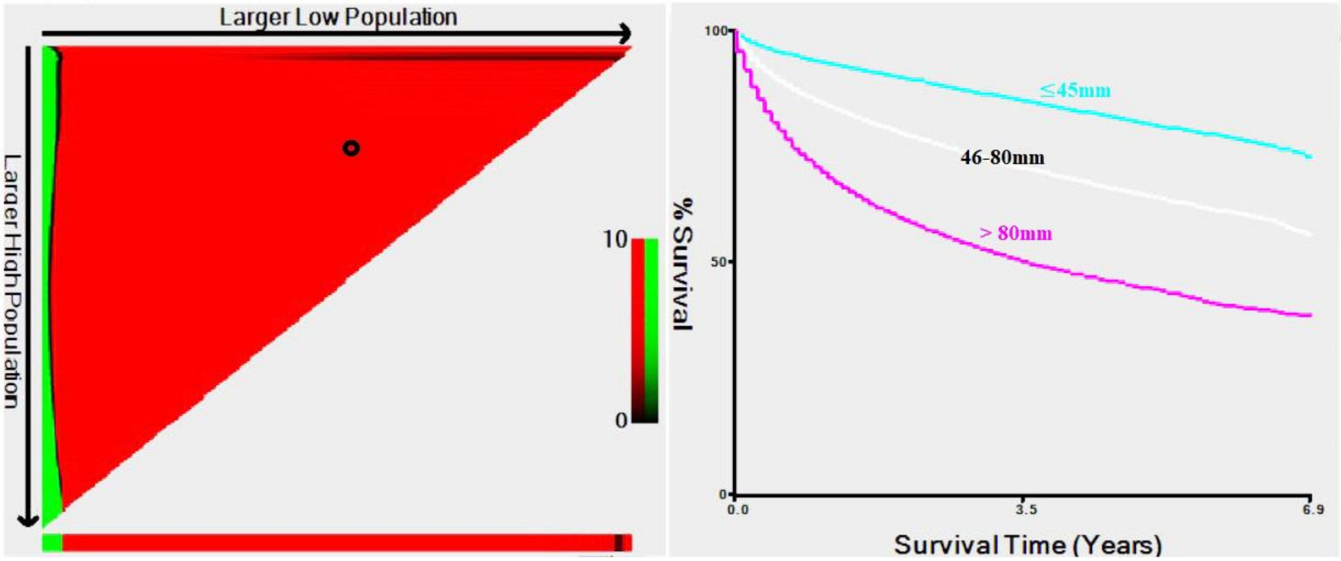

Supplementary Figure 1. Estimation of the cutoff value for tumor size stratification, as determined by X-tile software.
