# Supplementary material for: Clinical features and prognostic factors of patients with metastatic renal cell carcinoma stratified by age
Source: Aging (Albany NY). 2021 Mar 3;13(6):8290–305. doi: 10.18632/aging.202637 (PMC8034891; doi:10.18632/aging.202637)
Supplement: Supplementary Tables [file aging-13-202637-s002.pdf]

SUPPLEMENTARY TABLES

Supplementary Table 1. Clinical characteristics of renal cell carcinoma with and without distant metastasis.

		Total	%/SD	M0	%/SD	M1	%/SD	P-value
		n=79060	100.0%	n=68207	100.0%	n=10853	100.0%	
**Age**	Year	62.9	13.1	62.4	13.0	65.6	12.8	<0.001
**Tumor size**	mm	5.5	6.6	5.0	6.2	9.0	8.0	<0.001
**Marital status**	Married	46455	58.8%	40388	59.2%	6067	55.9%	<0.001
	Unmarried	28178	35.6%	23839	35.0%	4339	40.0%	
	Unknown	4427	5.6%	3980	5.8%	447	4.1%	
**Race**	White	64258	81.3%	55320	81.1%	8938	82.4%	<0.001
	Black	9912	12.5%	8775	12.9%	1137	10.5%	
	Other	4890	6.2%	4112	6.0%	778	7.2%	
**Sex**	Male	50569	64.0%	43211	63.4%	7358	67.8%	<0.001
	Female	28491	36.0%	24996	36.6%	3495	32.2%	
**Year of diagnosis**	2010-2012	37558	47.5%	32392	47.5%	5166	47.6%	0.420
	2013-2015	41502	52.5%	35815	52.5%	5687	52.4%	
**Grade**	I-II	35208	44.5%	34163	50.1%	1045	9.6%	<0.001
	III-IV	21057	26.6%	17673	25.9%	3384	31.2%	
	Unknown	22795	28.8%	16371	24.0%	6424	59.2%	
**Laterality**	Left	38526	48.7%	33241	48.7%	5285	48.7%	<0.001
	Right	39764	50.3%	34761	51.0%	5003	46.1%	
	Others	770	1.0%	205	0.3%	565	5.2%	
**T stage**	≤T1	51139	64.7%	49058	71.9%	2081	19.2%	<0.001
	T2	8162	10.3%	6422	9.4%	1740	16.0%	
	T3	13928	17.6%	10423	15.3%	3505	32.3%	
	T4	1996	2.5%	608	0.9%	1388	12.8%	
	TX	3835	4.9%	1696	2.5%	2139	19.7%	
**N stage**	N0	71777	90.8%	65743	96.4%	6034	55.6%	<0.001
	N1	4764	6.0%	1400	2.1%	3364	31.0%	
	NX	2519	3.2%	1064	1.6%	1455	13.4%	
**Surgery**	No/Unknown	14467	18.3%	7381	10.8%	7086	65.3%	<0.001
	Radical nephrectomy	30413	38.5%	27416	40.2%	2997	27.6%	
	Other operation	34180	43.2%	33410	49.0%	770	7.1%	
**Radiation**	No/Unknown	75838	95.9%	67888	99.5%	7950	73.3%	<0.001
	Yes	3222	4.1%	319	0.5%	2903	26.7%	
**Chemotherapy**	No/Unknown	72557	91.8%	66858	98.0%	5699	52.5%	<0.001
	Yes	6503	8.2%	1349	2.0%	5154	47.5%	
**Histological type**	Clear cell adenocarcinoma	42702	54.0%	38473	56.4%	4229	39.0%	<0.001
	Renal cell carcinoma	16990	21.5%	12756	18.7%	4234	39.0%	
	Papillary adenocarcinoma,	9426	11.9%	9003	13.2%	423	3.9%	
	Other	9942	12.6%	7975	11.7%	1967	18.1%	

Supplementary Table 2. Univariate and multivariate analysis of overall survival (OS) rates of patients with distant metastases.

		Univariate analysis		Multivariate analysis	
		HRs (95% CI)	P-value	HRs (95% CI)	P-value
**Marital status**	Married	1 (Ref)		1 (Ref)	
	Unmarried	1.30 (1.24-1.35)	<0.001	1.11 (1.06-1.16)	<0.001
	Unknown	0.99 (0.89-1.10)	0.798	0.91 (0.82-1.02)	0.105
**Age**	≤ 67 years	1 (Ref)		1 (Ref)	
	68-80 years	1.29 (1.23-1.35)	<0.001	1.22 (1.16-1.28)	<0.001
	> 80 years	2.12 (1.99-2.25)	<0.001	1.53 (1.43-1.63)	<0.001
**Race**	White	1 (Ref)		1 (Ref)	
	Black	1.15 (1.08-1.23)	<0.001	1.03 (0.96-1.11)	0.35
	Other	0.93 (0.86-1.01)	0.075	0.95 (0.87-1.03)	0.179
**Sex**	Male	1 (Ref)		1 (Ref)	
	Female	1.13 (1.08 -1.18)	<0.001	1.02 (0.97-1.06)	0.521
**Year of diagnosis**	2010-2012	1 (Ref)		1 (Ref)	
	2013-2015	0.94 (0.90-0.98)	0.007	0.93 (0.89-0.97)	0.001
**Histological grade**	I-II	1 (Ref)		1 (Ref)	
	III-IV	1.42 (1.30-1.54)	<0.001	1.44 (1.32-1.57)	<0.001
	Unknown	2.45 (2.27-2.66)	<0.001	1.26 (1.16-1.38)	<0.001
**Laterality**	Left	1 (Ref)		1 (Ref)	
	Right	0.99 (0.95 -1.04)	0.757	0.98 (0.94-1.03)	0.421
	Unknown	1.36 (1.24-1.49)	<0.001	0.96 (0.87-1.06)	0.428
**T stage**	≤T1	1 (Ref)		1 (Ref)	
	T2	1.09 (1.02-1.17)	0.017	1.04 (0.96-1.14)	0.328
	T3	0.87 (0.82-0.93)	<0.001	1.14 (1.05-1.23)	0.001
	T4	1.43 (1.33-1.54)	<0.001	1.25 (1.15-1.37)	<0.001
	TX	1.61 (1.51-1.72)	<0.001	1.09 (1.00-1.18)	0.047
**N stage**	N0	1 (Ref)		1 (Ref)	
	N1	1.69 (1.61-1.76)	<0.001	1.46 (1.39-1.53)	<0.001
	NX	1.69 (1.59-1.80)	<0.001	1.14 (1.06-1.22)	<0.001
**Surgery**	No/Unknown	1 (Ref)		1 (Ref)	
	Radical nephrectomy	0.37 (0.35-0.39)	<0.001	0.42 (0.39-0.46)	<0.001
	Other operation	0.34 (0.31-0.38)	<0.001	0.41 (0.37-0.45)	<0.001
**Radiation**	No/Unknown	1 (Ref)		1 (Ref)	
	Yes	0.99 (0.94-1.03)	0.527	1.00 (0.94-1.06)	0.924
**Chemotherapy**	No/Unknown	1 (Ref)		1 (Ref)	
	Yes	0.70 (0.67-0.73)	<0.001	0.64 (0.61-0.67)	<0.001
**Metastasis at bone**	No	1 (Ref)		1 (Ref)	
	Yes	1.18 (1.13-1.23)	<0.001	1.24 (1.18-1.30)	<0.001
**Metastasis at brain**	No	1 (Ref)		1 (Ref)	
	Yes	1.38 (1.30-1.47)	<0.001	1.38 (1.29-1.48)	<0.001
**Metastasis at liver**	No	1 (Ref)		1 (Ref)	
	Yes	1.71 (1.63-1.79)	<0.001	1.42 (1.35-1.50)	<0.001
**Metastasis at lung**	No	1 (Ref)		1 (Ref)	
	Yes	1.28 (1.22-1.33)	<0.001	1.32 (1.26-1.38)	<0.001
**Tumor size**	≤45mm	1 (Ref)		1 (Ref)	
	46-80mm	0.97 (0.91-1.04)	0.406	1.08 (1.01-1.16)	0.034
	> 80mm	1.03 (0.97-1.10)	0.344	1.18 (1.09-1.28)	<0.001
	Unknown	1.58 (1.46-1.71)	<0.001	1.08 (0.98-1.19)	0.109

Abbreviations: HR=Hazard ratio; CI=confidence interval.

Supplementary Table 3. Multivariate analysis of overall survival (OS) rates of patients with lung-related metastases.

		N=6589	HRs (95% CI)	P-value
**Marital status**	Married	3736	1 (Ref)	
	Unmarried	2586	1.09 (1.03-1.15)	0.003
	Unknown	267	0.88 (0.77-1.01)	0.072
**Age**	≤ 67 years	3828	1 (Ref)	
	68-80 years	1935	1.16 (1.09-1.23)	<0.001
	> 80 years	826	1.4 (1.33-1.58)	<0.001
**Race**	White	5416	1 (Ref)	
	Black	636	1.08 (0.99-1.18)	0.093
	Other	537	0.88 (0.80-0.98)	0.015
**Sex**	Male	4525	1 (Ref)	
	Female	2064	1.04 (0.98-1.10)	0.17
**Year of diagnosis**	2010-2012	3081	1 (Ref)	
	2013-2015	3508	0.95 (0.90-1.00)	0.044
**Histological grade**	I-II	584	1 (Ref)	
	III-IV	2101	1.37 (1.23-1.53)	<0.001
	Unknown	3904	1.24 (1.11-1.38)	<0.001
**Laterality**	Left	3223	1 (Ref)	
	Right	3107	0.95 (0.90-1.00)	0.073
	Unknown	259	1.05 (0.92-1.21)	0.462
**T stage**	≤T1	928	1 (Ref)	
	T2	1148	1.03 (0.92-1.15)	0.661
	T3	2336	1.11 (1.01-1.23)	0.037
	T4	915	1.22 (1.09-1.37)	<0.001
	TX	1262	1.05 (0.94-1.17)	0.393
**N stage**	N0	3536	1 (Ref)	
	N1	2121	1.38 (1.30-1.47)	<0.001
	NX	932	1.11 (1.02-1.21)	0.015
**Surgery**	No/Unknown	4440	1 (Ref)	
	Radical nephrectomy	1769	0.43 (0.39-0.47)	<0.001
	Other operation	380	0.43 (0.38-0.49)	<0.001
**Radiation**	No/Unknown	5111	1 (Ref)	
	Yes	1478	1.00 (0.93-1.09)	0.903
**Chemotherapy**	No/Unknown	3245	1 (Ref)	
	Yes	3344	0.59 (0.56-0.63)	<0.001
**Metastasis at bone**	No	4477	1 (Ref)	
	Yes	2112	1.26 (1.18-1.34)	<0.001
**Metastasis at brain**	No	5784	1 (Ref)	
	Yes	805	1.36 (1.25-1.49)	<0.001
**Metastasis at liver**	No	5144	1 (Ref)	
	Yes	1445	1.42 (1.34-1.52)	<0.001
**Tumor size**	≤45mm	677	1 (Ref)	
	46-80mm	1721	1.04 (0.94-1.15)	0.468
	> 80mm	3390	1.14 (1.03-1.27)	0.015
	Unknown	801	1.11 (0.98-1.26)	0.103

Abbreviations: HR=Hazard ratio; CI=confidence interval.

Supplementary Table 4. Multivariate Cox regression analysis of overall survival (OS) rates of the metastatic renal clear cell adenocarcinoma.

		n=4229	HRs (95% CI)	P-value
**Marital status**	Married	2613	1 (Ref)	
	Unmarried	1447	1.14 (1.05-1.23)	0.001
	Unknown	169	0.98 (0.81-1.19)	0.872
**Age**	≤ 67 years	2759	1 (Ref)	
	68-80 years	1201	1.29 (1.19-1.40)	<0.001
	> 80 years	269	1.65 (1.43-1.90)	<0.001
**Race**	White	3637	1 (Ref)	
	Black	280	1.15 (1.00-1.32)	0.057
	Other	312	0.97 (0.84-1.11)	0.615
**Sex**	Male	2945	1 (Ref)	
	Female	1284	1.10 (1.02-1.19)	0.016
**Year of diagnosis**	2010-2012	1933	1 (Ref)	
	2013-2015	2296	0.91 (0.85-0.98)	0.015
**Histological grade**	I-II	755	1 (Ref)	
	III-IV	1851	1.44 (1.29-1.61)	<0.001
	Unknown	1623	1.15 (1.03-1.29)	0.015
**Laterality**	Left	2101	1 (Ref)	
	Right	2012	0.97 (0.91-1.05)	0.483
	Other	116	1.02 (0.82-1.28)	0.838
**T stage**	≤T1	733	1 (Ref)	
	T2	714	0.95 (0.82-1.10)	0.525
	T3	1820	1.04 (0.91-1.19)	0.538
	T4	477	1.22 (1.04-1.42)	0.012
	TX	485	1.03 (0.88-1.21)	0.707
**N stage**	N0	2828	1 (Ref)	
	N1	988	1.60 (1.47-1.74)	<0.001
	NX	413	1.23 (1.08-1.39)	0.002
**Surgery**	No/Unknown	1906	1 (Ref)	
	Radical nephrectomy	1904	0.39 (0.35-0.44)	<0.001
	Other operation	419	0.39 (0.33-0.45)	<0.001
**Radiation**	No/Unknown	2921	1 (Ref)	
	Yes	1308	1.10 (0.99-1.21)	0.068
**Chemotherapy**	No/Unknown	1882	1 (Ref)	
	Yes	2347	0.72 (0.67-0.78)	<0.001
**Metastasis at bone**	No	2646	1 (Ref)	
	Yes	1583	1.30 (1.19-1.43)	<0.001
**Metastasis at brain**	No	3730	1 (Ref)	
	Yes	499	1.45 (1.29-1.63)	<0.001
**Metastasis at liver**	No	3577	1 (Ref)	
	Yes	652	1.43 (1.30-1.57)	<0.001
**Metastasis at lung**	No	1600	1 (Ref)	
	Yes	2629	1.44 (1.33-1.57)	<0.001
**Tumor size**	≤45mm	500	1 (Ref)	
	46-80mm	1288	1.15 (1.01-1.32)	0.039
	> 80mm	2160	1.25 (1.08-1.44)	0.003
	Unknown	281	1.26 (1.03-1.54)	0.023

Abbreviations: HR=Hazard ratio; CI=confidence interval.
